# In vitro efficacy of aqueous PVP-iodine solution below 5% as alternative to preoperative antisepsis in ophthalmology as the basis for an in vivo study

**DOI:** 10.1186/s12348-025-00489-3

**Published:** 2025-04-02

**Authors:** Paula Zwicker, Nevin Opitz, Julia Harris, Andreas Stahl, Ulrich Kellner, Ruth Koelb-Keerl, Philipp S. Muether, Anne Hunold, Axel Kramer

**Affiliations:** 1 Institute of Hygiene and Environmental Medicine, University Medicine Greifswald, 17475 Greifswald, Germany; 2 Section Antiseptic Stewardship of the German Society of Hospital Hygiene, Berlin, Germany; 3 Vereinigung operierender Augenärzte Nordrhein e.V. Dülkenstrasse 9, 51143 Köln, Germany; 4 Department of Ophthalmology, University Medicine Greifswald, 17475 Greifswald, Germany

**Keywords:** PVP-iodine, Antiseptic, Antimicrobial efficacy, Intravitreal injections

## Abstract

**Purpose:**

Pre-operative antisepsis of the conjunctiva is indicated prior to intraocular surgery to prevent post-interventional endophthalmitis. In Germany, antisepsis with 5% povidone-iodine (PI) aqueous solution is explicitly required prior to intravitreal injections (IVI), and also generally recommended for intraocular surgery. However, this concentration often leads to a foreign body sensation and an unpleasant burning in combination with dry eye symptoms. Postoperative eye pain, persistent corneal epithelial defects, and a risk of keratitis are further side effects. Due to the repetitive nature of IVI, these symptoms are particularly present in IVI patients. A reduced concentration may be favorable to decrease patient discomfort. A 1.25% PI solution does not increase the iodine concentration in the aqueous humor and is also used for prophylaxis of ophthalmia neonatorum and for preoperative antisepsis; in both cases the renal iodine excretion stays in a physiological range thus thyroid diseases are no contraindication for its use. Thus, the efficacy of reduced concentrations of PI should be evaluated in vitro.

**Methods:**

PI with dilutions below 5% (0.625 − 2.5% serial 1:2 dilution) was tested in vitro in a quantitative suspension assay and in a quantitative carrier test without and with addition of matrices to identify their antimicrobial effect against *Staphylococcus epidermidis*, *Pseudomonas aeruginosa*, *Cutibacterium acnes* and *Candida albicans*.

**Results:**

No differences in the antimicrobial effect was seen due to reduced concentrations of PI in comparison to a 5% solution. However, a trend was seen regarding the required contact time of the antiseptic solution.

**Conclusion:**

The in-vitro tests have shown adequate antisepsis of 1.25% PI prior to intraocular surgery. However, it is important to pay attention to a sufficient contact time of the antiseptic of about 1 min before ophthalmologic intervention. In order to give final recommendations, in vivo testing is needed to build a robust data foundation.

**Supplementary Information:**

The online version contains supplementary material available at 10.1186/s12348-025-00489-3.

## Highlights

5% PVP-Iodine solution prior IVI leads to unpleasant side effects.

In vitro, antimicrobial effect of 5% PI is comparable to that of 1.25% PI.

Addition of matrices had no visible effects on antimicrobial activity.

Contact time of 1 min is necessary.

**Supplementary Information:**

The online version contains supplementary material available at 10.1186/s12348-025-00489-3.

## Introduction

Periocular antisepsis, as well as antisepsis of the conjunctiva is indicated prior to any intraocular surgery to reduce the morbidity of post-interventional endophthalmitis [1]. Intravitreal injections (IVI) represent one of the most common ophthalmic surgeries besides cataract surgery and laser capsulotomy.

In order to reduce and prevent post-interventional endophthalmitis, several precautionary steps are recommended in addition to antisepsis using Povidone-iodine (PI).

The European Society of Cataract and Refractive Surgeons (ESCRS) in its current draft version of the Guideline for Cataract Surgery [2] recommends intracameral application of antibiotics “(e.g. cefuroxime 1 mg in 0.1 ml.) at the end of the cataract surgery to lower the risk for postoperative endophthalmitis. (GRADE +++)“ additionally to antisepsis. In its previous publication, the ESCRS [3] recommends applying PI 5%-10% to the cornea, conjunctival sac, and periocular skin for a duration of minimum 3 min prior to surgery, whereas the current draft version of the Cataract Guidelines recommends a regimen of 5–10% drops of PI 3 minutes before commencing cataract surgery or by continuously applying 0.25% povidone-iodine drops to wash the ocular surface every 20–30 seconds during the procedure.

PI is regarded as the most effective antiseptic; associated with significant reduction in ocular surface bacterial counts and significant decrease of post-interventional endophthalmitis [1, 4].

There are several recommendations in Germany that address the minimization of endophthalmitis risk, that not only include peri-operative antisepsis, but also other preventive measures. The joint recommendation of the German Ophthalmological Society, the Retinological Society and the Professional Association of Ophthalmologists in Germany includes additional measures to minimize endophthalmitis risk: performance of intravitreal injection under aseptic conditions, surgical hand antisepsis of staff, sterile gloves, sterile covers/incision foil, as well as a sterile speculum if used. Pre-operative antisepsis with 5% povidone-iodine aqueous solution (PI) in preparation of intravitreal injection [5] is recommended and it is mandatory within the German statutory Health Insurance (SHI) setting [6]. Analogously the guideline for prophylaxis and therapy of endophthalmitis of the German-speaking Society for Intraocular Lens Implantation and Refractive Surgery recommends 5% PI prior to any ophthalmic surgery, especially intraocular surgeries, with the comment that the optimal application concentration is open [7]. Even IVI of PI followed by vitrectomy was thought to be a safe and effective treatment for endophthalmitis [8, 9].

Mainly based on two studies, 5% PI is the most applied concentration, but there is no universal consensus on the concentration and exposure time [10], because the influence of concentrations below 5% PI on the incidence of endophthalmitis has not been extensively clinically investigated.

However, the 5% PI concentration often leads to a foreign body sensation and an unpleasant burning in combination with dry eye symptoms since PI acts as an extrinsic factor to dysregulate the homeostasis of a normal tear film. When applied to the ocular surface, PI triggers the inflammatory cascade of dry eye, potentially causing the patient to have worsening symptoms of their baseline dry eye disease. After repeated application prior to IVI, a significantly greater proportion of study eyes had a *Schein dry eye questionnaire score* of ≥ 7 as reported by patients [11]. Because intravitreal anti-VEGF injections had no effect on ocular surface, corneal endothelium, and anterior segment parameters [12], the 5% PI is likely to be the cause of dry eye. Infrequently, PI has been associated with complications including postoperative eye pain, persistent corneal epithelial defects, and risk of keratitis [13]. With anterior chamber entry of 5% PI side effects are to be expected [14], whereas local application of 1.25% PI does not increase the iodine concentration in the aqueous humor [15]. Furthermore, when using 1.25% PI for prophylaxis of ophthalmia neonatorum, the renal iodine excretion as well as the TSH level stay in a physiologic range [16]. Also, preoperative antisepsis with 1.25% has no influence regarding the renal iodine excretion; thus, thyroid diseases are no contraindication for application of 1.25% PI on the eye [13]. In animals PI ≥ 2% retards wound healing significantly [17], while 1.25% is tolerated even by the sensitive nasociliar epithelium [18]. When reducing the PI concentration below 5%, the risk for irritant contact dermatitis arising in 40% of patch tests with PI might also be reduced [19]. Since irritating effects of PI > 1% may lead to misperception as allergic contact dermatitis [20], reduction to 1.25% or below may have additional positive effects. As a result, reducing patient discomfort may be beneficial to keep patients in a continuous treatment cycle, which is often needed to prevent loss of vision. This is why a reduction of the PVP-I concentration for antisepsis of the conjunctiva prior IVI might be valuable.

Since 1.25% PI for ocular antisepsis seems to be ideal in terms of tolerability, PI with dilutions below 5% should be tested in vitro in a quantitative suspension assay and in a quantitative carrier test without and with addition of matrices relevant for the eye to guarantee its efficacy against Coagulase-negative staphylococci (CNS), *Cutibacterium acnes*, *Pseudomonas (P.) aeruginosa* and *Candida* (C.) *albicans* to ensure its efficacy. In the presented study with focus on 1.25% PI, CNS were selected as test organisms, because they are the most common bacteria on the lid margins and conjunctiva [21]. *Cutibacterium acnes*, a Gram-positive anaerobe representative of the deep resident bacterial skin flora, is more difficult to reach by skin antiseptics than Gram-positive bacteria of the superficial resident skin flora [22] and in rare cases can cause severe chronic endophthalmitis, characterized by slowly progressive intraocular inflammation which is typically not diagnosed until months after cataract surgery [23]. *P. aeruginosa* made up the largest proportion of bacteria related to endophthalmitis caused by Gram-negative bacteria [24] with very poor visual prognosis [25]. Endogenous fungal endophthalmitis is usually caused by *Candida spp.*, particularly *Candida* (*C.) albicans*, with increasing number of cases after intravitreal injections of anti-vascular endothelial growth factor treatment [26, 27].

## Methods

### PI solution, neutralizing solution and matrices

The test solutions were produced as given by DAC NRF 15.13 as far as possible (Supplement Table S1). Solutions with lower or higher concentrations were produced accordingly.

A solution with 0.3 g/l bovine serum albumin (BSA) was prepared using 30% BSA (Carl Roth, Karlsruhe, Germany) in sterile water. Cationorm MD sine^®^ and Biolan^®^ were used as matrix mimicking tear fluid.

Activity of PI was neutralized by addition of neutralizing solution (TLH-thio), containing 3% tween 80, 0.3% lecithin, 0.3% l-histidine, and 0.5% sodium thiosulfate after the exposure time.

### Composition of bacterial cultivation media

TSA plates were prepared using CASO agar (Carl Roth, Karlsruhe, Germany) as given by the manufacturer. Composition of YM-Medium and PYG agar are given in Table S2. *Cutibacterium acnes* was incubated in anaerobic conditions.

### Preparation of bacterial and fungal suspension

*S. epidermidis DSM 1798 / ATCC 12228 resp. P. aeruginosa DSM 939 / ATCC 15442*) were inoculated on TSA plates and incubated at 37 °C for 24 h. *C. albicans* was inoculated on YM Medium. For cultivation of *Cutibacterium acnes*, PYG was used.

Afterwards, bacteria were resuspended with 2 ml diluting solution (8.5 g NaCl, 1 g tryptone ad 1 l distilled water, trp/NaCl). The bacteria were washed three times with trp/NaCl following centrifugation at 10,000xg for 1 min and final centrifugation at 7,150xg for 3 min. Then the optical density (OD) of the suspension was adjusted to 0.17–0.18 (0.3-0.35 for *C. albicans*) at 630 nm being equivalent to 1-5 × 10^8^ colony-forming units (cfu)/ml. The suspension was diluted 7x in trp/NaCl and the three highest dilutions were plated on TSA plates to determine the colony forming units (cfu) of the initial solution.

### Quantitative suspension assay

The quantitative suspension assay was conducted as given by EN1040 but in a miniaturized assay using *S. epidermidis* and *P. aeruginosa*. First, 100 µl water or 0.3 g/l BSA solution were added in an autotube vial and 100 µl bacterial suspension with 1-5 × 10^8^ cfu/ml were mixed and incubated for 2 min following addition of 800 µl PI solution.

When using matrices mimicking tear fluid in the quantitative suspension assay, volumina were adjusted as following: 800 µl of artificial tear fluid were mixed with 100 µl bacterial suspension and after incubation for 2 min, 100 µl 10x PI solution were added.

This mixture was incubated for 30 s, 1 min and 3 min. Then, 100 µl were diluted in 800 µl neutralizing solution and 100 µl water. After 5 min incubation, the suspension was further diluted in dilution solution if necessary. As control of the test conditions, bacteria were not exposed to PI solution but to dilution solution instead.

The neutralized suspension was plated on TSA plates (100 µl) in duplicates and incubated for 24 h (48 h for *Candida albicans*) until counting of cfu. Next to the control of the test conditions, the efficacy of the neutralizing solution (control of neutralization) and validation of the procedure was done with a bacterial concentration of 1-5 × 10^4^ cfu/ml; 800 µl of the neutralizing solution were mixed with 100 µl dilution solution of test solution (PI) and after 5 min incubation, 100 µl bacteria solution were added. After further incubation for 3 min, the bacteria suspension was diluted and plated on TSA plates.

After incubation for 24 h, cfu on the agar plates were counted and the reduction was calculated according formula (1) where *n*_*c*_ is the number of cfu in the initial solution and *n*_*p*_ is the number of cfu in PI treated solution

1 $$Reduction = lo{g_{10}}({n_c}) - lo{g_{10}}\left( {{n_p}} \right)$$

According to EN 1040, samples with less than 14 cfu in sum (duplicates) on TSA plates after neutralization were included in the calculation with 14 cfu. Samples with more than 330 cfu per TSA plate were not considered, since further dilutions were made for all samples.

### Quantitative carrier test

Sterile steel carriers of stainless steel (20 mm in diameter, polished to grade 2) were loaded with 100 µl bacteria/fungal suspension (1-5 × 10^8^ cfu/ml) of *S. epidermidis*, *P. aeruginosa*, *Cutibacterium acnes* and *C. albicans* in dilution solution, BSA solution or Biolan^®^ that was spread with an inoculation loop. The suspension was dried for max. 30 min under sterile conditions on a heating plate (37 °C). *P. aeruginosa*, *Cutibacterium acnes* and *C. albicans* were dried until a thin liquid film remained. The *S. epidermidis* suspension was dried completely.

Then, 100 µl of PI were added and incubated for 30 s, 1–3 min (*S. epidermidis*, *P. aeruginosa*) following transfer in a 6-well plate containing 3 ml inactivator solution and glass beads. *C. albicans* and *Cutibacterium acnes* were incubated with PI for 1 min.

When shaking for 1 min, bacteria were resuspended. After incubation for further 4 min to neutralize iodine, the suspension was further diluted if necessary and the samples were plated on TSA, YM or PYG plates in duplicates. Agar plates were incubated for 24 h at 37 °C. As control, water instead of PI was used.

After incubation, the evaluation was made as described for the suspension assay.

### Statistical analysis

For comparison of reduction regarding contact time and PI concentration, a one-way ANOVA with Tukey’s multiple comparison was used.

## Results

**Fig. 1:**
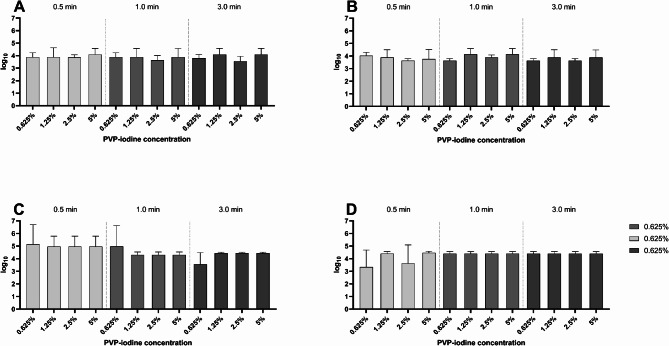
Lg reduction in the quantitative suspension assay without matrix or with protein-containing matrix. (**A**) *S. epidermidis*, without matrix. (**B**) *S. epidermidis* with 0.3 g/l BSA, (**C**) *P. aeruginosa* without matrix, (**D**) *P. aeruginosa* with 0.3 g/l BSA

**Fig. 2:**
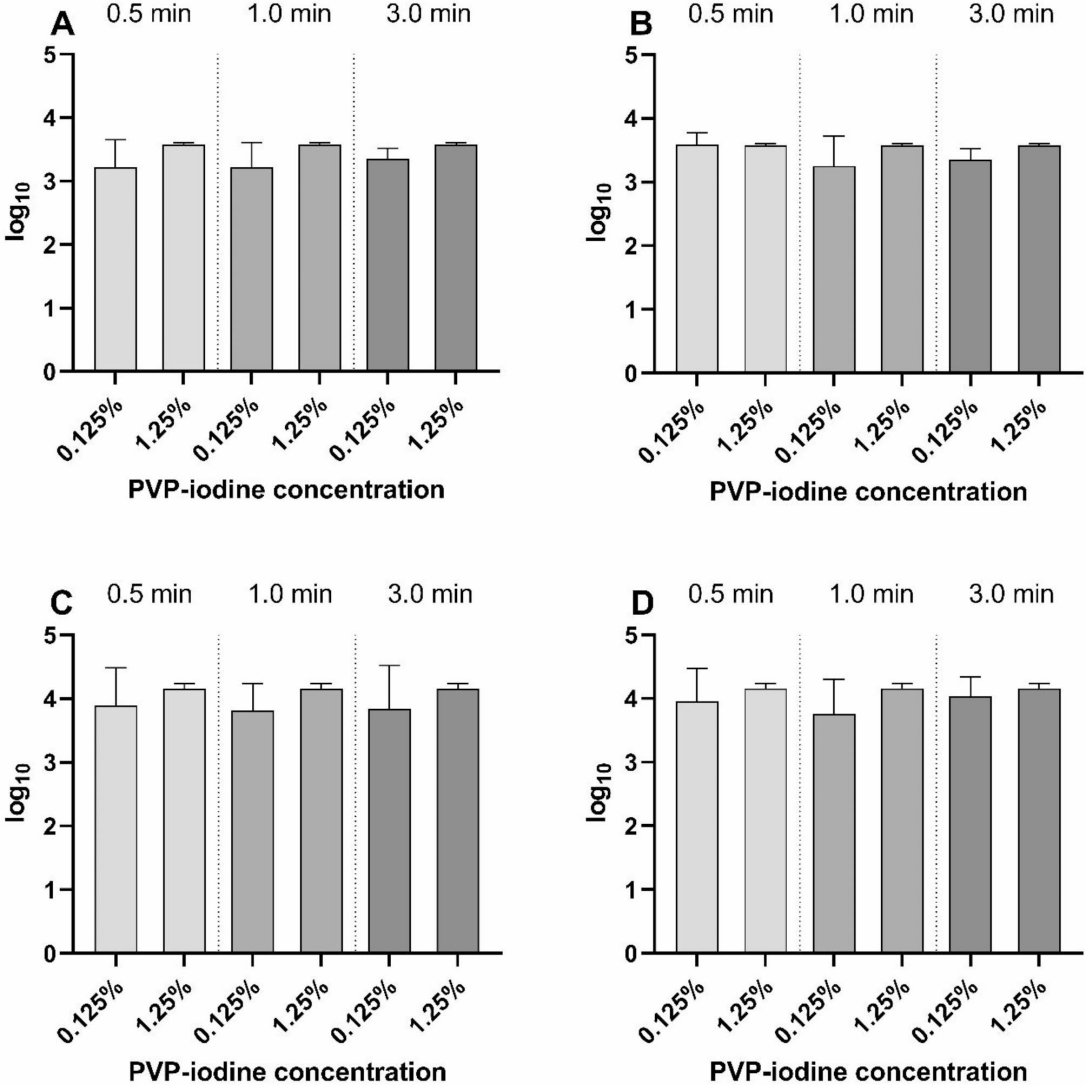
Lg reduction in the quantitative suspension assay with matrices mimicking tear fluid. (**A**) *S. epidermidis* in Cationorm MD sine^®^. (**B**) *S. epidermidis* in Biolan^®^, (**C**) *P. aeruginosa* in Cationorm, (**D**) *P. aeruginosa* in Biolan

In the quantitative suspension assay, the reduction shown for *S. epidermidis* and *P. aeruginosa* (Fig. 1) in sodium chloride solution reached the maximum of approximately 4 lg reduction that can be shown within the test setup. There were no statistically significant differences between the contact times (0.5 min, 1 min, 3 min) and between the used concentrations (0.625%, 1.25%, 2.5%, 5%). Similar results were obtained for the protein-containing matrix (0.3 g/l BSA). When using the eye drops Cationorm MD sine^®^ or Biolan^®^ as matrices, for both test organisms the reduction was about 3–4 lg levels for PI concentrations of 0.125% and 1.25% and contact times of 0.5 min, 1 min, and 3 min (Fig. 2).

In the quantitative carrier test, drying of the bacterial suspension resulted in a lg-reduction of approximately 1.71–2.71 lg levels (*S. epidermidis*) or 1.65–3.34 lg levels (*P. aeruginosa*), respectively. The reduction of *S. epidermidis* (Table 1) and *P. aeruginosa* (Table 2) due to PI reached the maximum that can be shown within the test setup of 2.86–5.32 lg for *S. epidermidis* and 3.4–7.83 lg fo*r P. aeruginosa*. Comparing the lg reduction of 1.25% PI with 5% PI solution, no statistically significant differences were observed between concentrations and exposure times.

**Table 1:** Bacterial count (lg levels) and Lg reduction of *S. epidermidis* treated with PI (1.25%, 2.5%, 5%) for 0.5 min, 1–3 min in a quantitative carrier test, *n* = 3

		NaCl	0.3 g/l BSA	Biolan^®^
Initial bacterial count		6.34 ± 0.30	6.63 ± 0.08	6.46 ± 0.16
		lg reduction		
1.25%	0.5 min	5.04 ± 0.39	2.94 ± 0.18	5.32 ± 0.15
	1.0 min	4.87 ± 0.34	3.26 ± 0.77	5.32 ± 0.15
	3.0 min	5.08 ± 0.16	5.01 ± 0.45	5.32 ± 0.15
2.50%	0.5 min	4.88 ± 0.82	3.22 ± 0.36	5.32 ± 0.15
	1.0 min	5.17 ± 0.32	3.50 ± 0.22	5.15 ± 0.32
	3.0 min	5.19 ± 0.29	4.96 ± 0.65	5.32 ± 0.15
5.00%	0.5 min	4.88 ± 0.64	2.86 ± 0.52	5.32 ± 0.15
	1.0 min	5.17 ± 0.34	3.40 ± 0.90	5.09 ± 0.10
	3.0 min	5.19 ± 0.29	4.10 ± 0.08	5.32 ± 0.15
Control*		2.71 ± 0.57	1.71 ± 0.22	2.48 ± 0.31

* Due to drying of the germs on the carrier, loss of cell viability occurred. Lg-reduction after PI treatment was calculated using the bacteria concentration of the initial suspension

**Table 2:** Bacterial count (lg levels) and Lg reduction of *P. aeruginosa* treated with PI (1.25%, 2.5%, 5%) for 0.5 min, 1–3 min in a quantitative carrier test, *n* = 3–6

		NaCl	0.3 g/l BSA	Biolan^®^
Initial bacterial count		7.11 ± 0.14	7.09 ± 0.20	7.14 ± 0.12
		lg reduction		
1.25%	0.5 min	5.42 ± 0.73	3.40 ± 0.54	5,26 ± 1,18
	1.0 min	5.56 ± 0.84	3.84 ± 0.37	5,97 ± 0,70
	3.0 min	5.86 ± 0.37	4.80 ± 0.61	5,97 ± 0,13
2.50%	0.5 min	5.26 ± 0.72	4.02 ± 0.57	5,61 ± 0,59
	1.0 min	5.30 ± 0.26	4.36 ± 0.26	5,52 ± 0,64
	3.0 min	5.92 ± 0.26	5.82 ± 0.30	5,90 ± 0,15
5.00%	0.5 min	7.83 ± 0.54	3.97 ± 0.51	5,03 ± 0,92
	1.0 min	5.90 ± 0.25	4.33 ± 1.06	5,58 ± 0,65
	3.0 min	5.71 ± 0.63	4.82 ± 1.18	5,91 ± 0,14
Control		3.29 ± 0.14	1.65 ± 0.17	3.34 ± 1.53

For *C. albicans*, a reduction of > 4 lg was reached within a contact time of 1 min in the quantitative carrier test when using 0.3 g/l BSA as a matrix (Table 3). There were no differences between the effectivity of 1.25% and 5.00% PI. Similar results were achieved for *Cutibacterium acnes* with a reduction of > 5 lg levels within 1 min.

**Table 3:** Lg reduction of *C. albicans* and *Cutibacterium acnes *treated with PI (1.25%, 5%) for 1 min in a quantitative carrier test with addition of 0.3 g/l BSA, *n* = 3

		Candida albicans	Cutibacterium acnes
**Initial bacterial count**, **cfu**		6.47 ± 0.26	8.00 ± 0.58
**Lg reduction**	1.25%	4.32 ± 0.25	5.85 ± 0.57
	5.00%	4.32 ± 0.25	5.85 ± 0.57
	Control	1.15 ± 0.17	1.25 ± 0.49

## Discussion

To reduce the risk of infection, PI rinsing before any intraocular surgery is indicated, whereby a concentration of 5% is mainly used. Since IVI treatment is often needed frequently with multiple IVI p.a., especially in patients with neovascular age related macular degeneration (commonly referred to as wet AMD), conjunctival and/or corneal irritation with dry eye symptoms may lead to a reduced patient adherence/persistance [28, 29]. A reduced PI concentration therefore would be helpful to maintain patient adherence. At the same time, lower PI concentrations are only suitable if endophthalmitis rates are not increased.

Endophthalmitis incidences following IVI range between 0.028% [30] and 0.036% [31]. Dossarps et al. reported 65 cases of presumed endophthalmitis in 316,576 IVI resulting in an overall incidence of 0.021% [32].

Several publications regarding low concentrated PI solutions for use in regard with eye surgery exist. Peden et al. and Shimada et al. both tested PI with low concentrations (0.25–1.25%) as antiseptic; however in both studies the number of cases is too low to detect effects on the incidence of endophthalmitis or the incidence using diluted PI is higher than for 5% PI [33, 34]. However, when using 0.25% PI before and after insertion of the lid speculum and again after the injection, in addition with post-surgery antibiotic administration, no endophthalmitis cases occur after 15,144 IVI [35]. These data are indeed attractive but hardly to compare to the routine procedure given in different guidelines. Some other publications use diluted PI solutions; however they were applied as a treatment procedure to endophthalmitis patients and not as prophylactic measure [8, 36, 37]. Furthermore, none of these studies has a secondary objective regarding dry eye symptoms next to endophthalmitis rates.

Oliverio et al. stated an improvement in dry eye symptoms due to treatment with 0.6% PI in a nanoemulsion [38]. An in vitro study using human corneal epithelial cells treated with a maximum of 50,000 ppm (5%) PI revealed increasing cytotoxicity starting with a concentration of 0.1% [39]. These data suggest that a lower PI concentration will have positive effects on side effects as dry eyes.

Regarding the bacterial load, Shimada et al. published interesting work after rinsing the eye every 20–30 s during a cataract surgery with a PI solution leading to a significant reduction of bacterial contamination [40]. Similar results were presented by Reibaldi et al. who showed significant reduction of bacteria in swabs of about 82% (1.91 lg levels) after 3 day prophylactic treatment of the eye with 0.6% PI [41]. In vitro, a 0.6% PI solution had antibacterial effects after 5 min against *S. aureus*, *S. epidermidis* and *P. aeruginosa* [42]. However, *Candida*. were susceptible towards PI only after 24 h of contact.

In the presented study, without and with matrices, all tested concentrations reached a 3 lg level reduction for bacteria and *C. albicans*, which is required for antiseptics [43] within the tested exposure times. The high efficiency of lower concentrated PI may result from a higher concentration of free iodine in lower concentrated PI [44]. Thus, reduction of concentration up to 1.25% also considering the load with inorganic matrix on the eye surface does not lead to reduced efficacy, which supports the use of lower concentrations of PI than 5%. However, a trend was seen between the contact times when adding 0.3 g/l BSA as matrix. Thus, it is important to pay attention to a sufficient contact time of the antiseptic of about 1 min prior to ophthalmologic intervention.

However, there are some limitations of the study: Only planktonic bacteria were used and the efficacy of PI against bacteria in biofilms is lacking. Furthermore, realistic conditions are not displayed by the use of eye drops because of the special composition of the tear film. By use of protein matrices, however, a worst-case scenario is created.

Based on the results, a concentration of 1.25% is sufficient for eye antisepsis. This is in agreement with the fact that a significant reduction of the conjunctival flora is also achieved in vivo by 1.25% PI [45] when using it for prophylaxis of *Ophthalmia neonatorum*. Silas et al. [46] stated that even a 1% PI solution may be used for preoperative antisepsis when applying 3x for 30 s. In contrast, Ferguson et al. [47] compared the efficacy of 1% and 5% PI with the result that 5% PI is more efficient on bacterial reduction when irrigating the fornix conjunctivae particularly in the presence of heavier initial bacterial load [47].

With regard to ophthalmological interventions, also viruses, especially adenoviruses and their inactivation are of importance. Eggers et al. have shown that diluted PI (1%) is efficient in inactivation of modified vaccinia virus Ankara (MVA) within 15 s in clean and dirty conditions (> 4 lg inactivation) [48] confirming its activity against enveloped viruses. However, regarding adenoviruses, disinfectants with *limited spectrum of virucidal activity* according to EN standards are necessary. For that, disinfectants are tested using adenovirus type 5. Results of Wada et al. who used adenovirus type 3 are promising since they revealed virucidal activity (> 4 lg inactivation) of a 1% PI solution within 5 min and of a 0.1% PI solution within 1 min [49]. New formulations using iodine immobilized e.g. in polymers and nanoparticles [50] may lead to even better tolerability.

Another important point when using antiseptics is the development of tolerance and resistance towards antiseptic agents especially when using low concentrations. Regarding PI, a study of Craven et al. describes a colonization of iodine solution with *Pseudomonas sp* [51]. In addition, Coetzee et al. isolated PI resistant *P. aeruginosa* from burn injuries [52]. In contrast, several studies stated that a resistance to PI is unlikely [53–56]. Due to its mode of action, there is also no risk of developing tolerance or resistance even if the application concentration is reduced to 1.25%. Using electron microscopy and biochemical tests, it was shown that PI is interacting with the cell wall of microorganisms and thus leads to the formation of pores in the lipid membranes leading to leakage of cytosol [57]. Furthermore, enzymes are denatured by intracytoplasmic protein oxidation after penetration of free iodine through the cell membrane [58].

Before testing efficacy in vivo, the efficacy of 1.25% PI should be compared to the efficacy of 5% PI regarding inactivation of adenovirus type 5 in vitro. In continuation of the in-vitro results, efficacy and tolerability have to be tested in vivo. In this study, concentration, contact time, but also delivery application (rinsing vs. topical application) have to be taken into consideration when testing efficacy. Furthermore, tolerability should be examined. It is necessary to examine in vivo side effects such as dry eyes when using reduced PI concentrations and to exclude effects on the endophthalmitis rate after IVI when using lower PI concentrations. A reduction of PI concentration is only conceivable with consistent antimicrobial activity of the antiseptic agent; means with no increase in endophthalmitis rate. In all cases, a uniform approach to antisepsis as prescribed by some professional societies as described above should be used to generate reliable data.

## Conclusion

In the current study, we proved 1.25% PI to be as efficient in bacteria/fungi reduction as 5% PI. Based on these data, and considering the literature, the efficiency and tolerability of 1.25% PI need be investigated in vivo. In a follow-up study, including different contact times and delivery methods, efficacy needs to be proven. If it is found to be equivalent to 5% PI, the next step is to clinically verify its equivalence regarding prevention of side effects after intravitreal injections in a double-blinded randomized controlled study. A reduction of the PI concentration from 5 to 1.25% may effectively reduce patient discomfort, thus potentially increasing IVI patient adherence and persistence to continuous treatment.

## Electronic Supplementary Material

Below is the link to the electronic supplementary material.

Supplementary Material 1

## Data Availability

The datasets used and/or analysed during the current study are available from the corresponding author on reasonable request.
