# Supplementary material for: In vitro efficacy of aqueous PVP-iodine solution below 5% as alternative to preoperative antisepsis in ophthalmology as the basis for an in vivo study
Source: J Ophthalmic Inflamm Infect. 2025 Apr 2;15:35. doi: 10.1186/s12348-025-00489-3 (PMC11965046; doi:10.1186/s12348-025-00489-3)
Supplement: Supplementary file 1 — Supplementary Material 1 [file 12348_2025_489_MOESM1_ESM.docx]

**Supplement**

Table S1 Composition of PI solutions

| PI concentration | 0.625% | 1.25% | 2.5% | 5.0% | 12.5% |
| --- | --- | --- | --- | --- | --- |
| PI | 0.625 g | 1.25 g | 2.5 g | 5.0 g | 12.5 g |
| Sodium chloride | 0.8 g | 0.8 g | 0.7 g | 0.5 g | 0.08 g |
| Disodium phosphate solution 1.25%, pH 8.5-9.5 | 10.0 g | 20.0 g | 40.0 g | 80.0 g | 2.0 g |
| Water | Ad 100 g | | | | |

Table S2 Composition of cultivation media

| Medium, constituent | amount |
| --- | --- |
| **YM medium** |  |
| Yeast extract | 0.3% |
| Malt extract | 0.3% |
| Peptone from sy beans | 0.5% |
| glucose | 1.0% |
| **PYG agar** |  |
| Peptone from casein | 0.5% |
| Peptone from soy beans | 0.5% |
| Yeast extract | 1.0% |
| Meat extract | 0.5% |
| glucose | 0.5% |
| K_2_HPO_4_ | 0.2% |
| Tween 80 | 0.1% |
| Resazurin | 1 mg/l |
| salt solution | 40 ml/l |
| Vitamin K1 solution | 0.2 ml/l |
| Haemin solution | 10 ml/l |
| Cysteine-HCl | 0.05% |
| **Salt solution** |  |
| CaCl_2_ x 2 H_2_O | 0.05% |
| MgSO_4_ x 7 H_2_O | 0.1% |
| K_2_HPO_4_ | 0.2% |
| KH_2_PO_4_ | 0.2% |
| NaHCO_3_ | 2% |
| NaCl | 0.4% |
